# Cryptogamic Biomass in Pannonic Acidic Sand Steppes Subject to Changing Land-Use

**DOI:** 10.3390/plants12162972

**Published:** 2023-08-17

**Authors:** Rebeka Aszalósné Balogh, Edit Farkas, Júlia Tüdősné Budai, László Lőkös, Gábor Matus

**Affiliations:** 1 Department of Applied Plant Biology, Institute of Crop Sciences, University of Debrecen, Böszörményi u. 138, 4032 Debrecen, Hungary; 2 Department of Botany, Institute of Biology and Ecology, Faculty of Sciences & Technology, University of Debrecen, Egyetem tér 1, 4032 Debrecen, Hungary; matus.gabor@science.unideb.hu; 3 Institute of Ecology and Botany, Centre for Ecological Research, Alkotmány u. 2-4, 2163 Vácrátót, Hungary; farkas.edit@ecolres.hu; 4 Research Institute of Karcag, Hungarian University of Agriculture and Life Sciences, Kisújszállási u. 166, 5300 Karcag, Hungary; budaijulia86@gmail.com; 5 Department of Botany, Hungarian Natural History Museum, Pf. 137, 1431 Budapest, Hungary; lokos.laszlo@nhmus.hu

**Keywords:** biomass, bryophytes, cryptogams, dry grassland, lichens

## Abstract

Cryptogams, often neglected in vegetation dynamics studies, compose a large part of biomass and contribute to the biodiversity of sandy grasslands. Since the work of Verseghy (1970s), their productivity has not been analyzed in Hungary. We studied the lichen and bryophyte dynamics (hereinafter called cryptogams) at two Eastern Hungarian dry sandy grassland sites. The sites of *Corynephorus canescens* and of *Festuca vaginata* dominance, respectively, belonging to the community *Festuco vaginatae–Corynephoretum* have been monitored. We aimed at (1) quantifying the diversity and biomass of the cryptogamic communities; (2) exploring the cryptogamic response to management changes; and (3) studying the effect of experimental management (fencing) on the cryptogamic assemblages. The sites have been compared in 2013 and 2018, respectively. Forty microplots per site per management have been analyzed in both years. Samples of lichens and bryophytes were hand-sorted, dried and then measured. Fencing has led to increased biomass of cryptogams within a few years. Lichens in general benefited comparatively more from exclosure than bryophytes. The increase in lichen biomass (especially that of *Cladonia rangiformis*) is clearly due to the over 10-year absence of grazing. The only lichen favored by moderate grazing is the legally protected *C. magyarica*. Short spells of low-intensity grazing can promote the species richness of cryptogams in the community.

## 1. Introduction

The diversity of terricolous lichens and bryophytes can be substantial, sometimes exceeding that of vascular plant species [1,2,3,4,5,6]. Globally, their number is much lower than that of the vascular plants, but despite their small size, their ecological and economic role is not negligible (bryophytes: [7,8,9,10,11,12], lichens: [13,14,15]). Although often neglected in the studies of vegetation dynamics, these two taxonomic groups can compose a large part of biomass and can largely contribute to the biodiversity in sandy grasslands.

Conservation efforts in European dry grasslands focus mainly on ‘keystone species’ of vascular plants, thus leaving several taxa (among them various cryptogams) almost overlooked [16,17,18,19,20]. Knowledge of the conservation status of bryophytes and lichens is still scarce, and usually their distribution is less well documented than that of the vascular plants. In the last 20 years, in the European dry habitats, numerous projects studied the biodiversity patterns and functional role of lichens and bryophytes [21,22,23,24,25,26,27,28,29,30,31,32]. Lichen- and bryophyte-rich dry habitats, in spite of being fragmented by anthropogenic activities, can still reach a rather large extent [33,34].

In lowlands, dry habitats with high cryptogamic diversity occur mostly on sand. Sandy grasslands, both on acidic and on calcareous substrata, are widely distributed throughout Europe. These grasslands, as a rule, are exploited as pastures, so their conservation status is highly dependent on management through livestock grazing [35,36,37,38].

Sandy grasslands of the Pannonic region (Pannonic and Pontic sandy steppes by EC Directorate-General for Environment et al. [39]) are threatened by plant invasions and changes in management intensity, including cessation [40]. In spite of this, only a few studies dealt with the effect of grazing (and its abandonment) on sandy grasslands [41,42]. Neither has their spontaneous dynamics been studied, and this especially holds for cryptogams.

Several studies, mostly from the tundra, alpine grasslands and heath communities, showed that significant changes occur in cryptogamic communities at the onset of grazing, during changes in grazing intensity [43,44,45,46,47,48,49,50,51] and during its cessation [52]. Consistent shifts in lichen community composition are also associated with grazing [53,54,55,56,57]. Grazing often reduces the cover and species richness of cryptogams and may also induce sharp shifts in composition [44,46,47,58,59]. Disturbance by grazing may finally lead to an alternative successional state (a novel cryptogamic ecosystem) [60]. Documentation of diversity differences between grazed and ungrazed areas is rather sparse and, where available, inconsistent [51,61,62].

Since the pioneering works of Verseghy [63,64,65,66,67], cryptogamic biomass and productivity have not been analyzed in Hungary, except for a recent study of the production of the lichen secondary metabolites of a few steppe species [31,32]. Phytosociological studies tend to neglect or discuss only partly the cryptogams. This also holds for Hungarian reviews of the open acidic sandy steppes (*Festuco vaginatae–Corynephoretum* and other communities) where cryptogams have not been covered [68] or only bryophytes have been discussed [69]. Cryptogams have recently been pooled also in studies of calcareous grasslands [70,71]. A recent positive exception is an analysis of secondary succession on Hungarian calcareous sands [72] where both lichens and bryophytes were covered specifically. Furthermore, microcoenological data on the composition of terricolous lichen communities related to their influencing factors were analyzed in calcareous grasslands by Veres et al. [30].

Methods of floristic and biomass survey in cryptogamic assemblages, due to their limited extension, differ significantly from those in vascular communities. A review by Berg et al. [73] demonstrated a large overall variation in the applied plot sizes, ranging from 0.0002 m^2^ to 30 m^2^, for lichen communities. The used plot sizes extend over five orders of magnitude, which is even more than those reported for relevés in vascular plant communities [74]. The most frequently used plot sizes were 0.04 and 0.01 m^2^, respectively. The size of the largest frequently used plot types has been 1 m^2^ and was mostly used for terricolous lichen communities, where vascular plants were also recorded [73,75]. Güler et al. [76] recommended that, wherever possible, plots of cryptogamic micro-communities should be square shaped (20 cm × 20 cm or 10 cm × 10 cm).

In the present study, we aimed (1) to quantify the floristic composition, diversity and biomass of terricolous cryptogamic communities; (2) to explore the response of cryptogams to management changes (subject to different grazing pressure); and (3) to study the effect of an experimental change (fencing) of management on the cryptogamic assemblages with regard to the performance of the legally protected lichen *Cladonia magyarica*.

## 2. Results

### 2.1. Species Richness

In total, 15 taxa (11 lichens and 4 bryophytes, 14 taxa from the *Corynephorus canescens* dominated site (CC) and 9 from the *Festuca vaginata* dominated site (FV), respectively) have been identified in the samples. The total number of lichen taxa tended to be higher in CC (up to nine at a given date and management) than in FV (three or four). The studied communities did not differ in the total number of bryophytes (three or four at a given date and management). 

All but one lichen species belong to the genus *Cladonia*. The only exception has been *Diploschistes muscorum*, a parasitic lichen growing on the thalli of *Cladonia* species.

The average numbers of lichens, bryophytes and all cryptogamic taxa are shown in Table 1, whereas detailed species lists, species frequencies and biomass distributions are available in Table A1 and Table A2 in Appendix A.

### 2.2. Composition

#### 2.2.1. Frequency

The total records of the taxa on the two dates, two communities and treatments were close to one thousand, out of which somewhat more than 40% came from the lichens and somewhat less than 60% from the bryophytes. All but one lichen species belong to the genus *Cladonia*, and this genus comprised more than 99.5% of all the lichen records too. The frequency of *Cladonia* taxa (occurrences out of the total of 320 records) has shown two magnitudes of variation. In both communities, the two most frequent species have been *C. rangiformis* (more than two-thirds of lichen records in total) and *C. rei* (about one-sixth of summed lichen records). The communities are distinguished by the presence of *C. foliacea* in the *Festuca vaginata* dominated site (<3% of all lichen records) and *C. furcata* and *C. magyarica* in the *Corynephorus canescens* dominated site (about 4% and 6.5% of all lichen records), respectively. Among the bryophytes, *Syntrichia ruralis* and *Brachythecium albicans* were more frequent, both having around 40%, while *Polytrichum piliferum* had around 20% of all bryophyte records (see also Table A1 and Table A2 in Appendix A).

#### 2.2.2. Biomass

**Figure 1:**
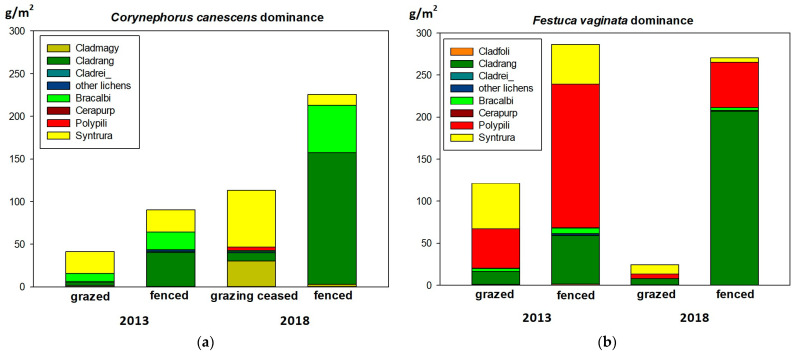
Estimates of specific cryptogamic biomass in the studied communities in 2013 and in 2018, respectively, from (**a**) *Corynephorus canescens* dominated site and (**b**) *Festuca vaginata* dominated site. Abbreviations of species names: Cladfoli—*Cladonia foliacea*, Cladmagy—*C. magyarica*, Cladrang—*C. rangiformis*, Cladrei_—*C. rei*, Bracalbi—*Brachythecium albicans*, Cerapurp—*Ceratodon purpureus*, Polypili—*Polytrichum piliferum*, Syntrura—*Syntrichia ruralis*.

The overall distribution of biomass among the lichens (46%) and bryophytes (54%) is similar to that of the overall frequency records. The averages of the biomass of the lichens, bryophytes and of all the cryptogamic taxa are shown in Table 2. The specific biomass data, however, differed greatly from the frequency data. The biomass of *Cladonia* taxa, forming the vast majority of the biomass, has shown four magnitudes of variation (from 0.01 g to almost 500 g). Of all the lichen biomass, *C. rangiformis* formed more than 90% (overrepresented compared to its frequency). In contrast, *C. foliacea*, *C. furcata* and *C. rei* have been strongly underrepresented compared to their frequencies (<1% of lichen biomass). The biomass share of *C. magyarica* has been similar to its frequency. Among the bryophytes, *Polytrichum* has been overrepresented (close to 45%), *Brachythecium* underrepresented, while *Syntrichia* had a consistent share compared to its frequency record (Figure 1) (see also Table A1 and Table A2 in Appendix A). 

**Figure 2:**
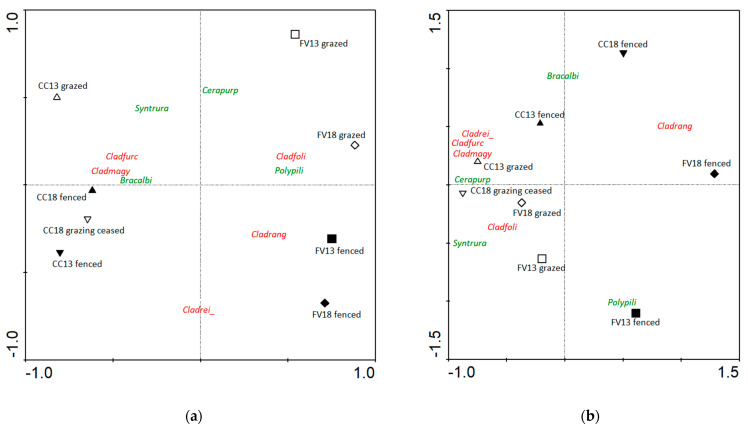
Species frequency (**a**) and biomass composition (**b**) of the studied sites, according to the principal component analysis (PCA). Explanation of symbols: empty up-triangle—CC13 grazed, filled up-triangle—CC13 fenced, empty down-triangle—CC18 grazed, filled down-triangle—CC18 grazing ceased, empty square—FV13 grazed, filled square—FV13 fenced, empty diamond—FV18 grazed, filled diamond—FV18 fenced. Scientific names are abbreviated to eight letters where the first four letters stand for the genus name while the last four ones for the species name, respectively. Bryophyte names are in green and lichen names are in red. Abbreviations of species names: Cladfoli—*Cladonia foliacea*, Cladfurc—*C. furcata*, Cladmagy—*C. magyarica*, Cladrang—*C. rangiformis*, Cladrei_—*C. rei*, Bracalbi—*Brachythecium albicans*, Cerapurp—*Ceratodon purpureus*, Polypili—*Polytrichum piliferum*, Syntrura—*Syntrichia ruralis*.

Principal coordinate analysis (PCA) clearly separated the *Corynephorus canescens* and *Festuca vaginata* dominated sites (by the *Y* axis), both in case of frequency and biomass data. When PCA was applied to the frequency records, it emphasized the dominance of small but widespread species such as *Cladonia magyarica* (CC) or *C. rei* (both sites) (Figure 2), while based on biomass records, it stressed vegetation dominants such as *Cladonia rangiformis* (both sites), *Brachythecium albicans* (CC) or *Polytrichum piliferum* (FV). The effect of the contrasting treatment is also clearly visible, as the fenced and grazed sites are shown separately on both sides of the *X* axis. Based on the frequency data, the CC ‘grazing ceased’ samples were positioned among the fenced samples, but when their biomass data have been considered, these were ordinated among the grazed ones. Irrespective of the data type, *Cladonia rangiformis* proved a good indicator of fenced stands, whereas grazed samples have been indicated by *Cladonia magyarica* and *Syntrichia ruralis* (Figure 2).

**Figure 3:**
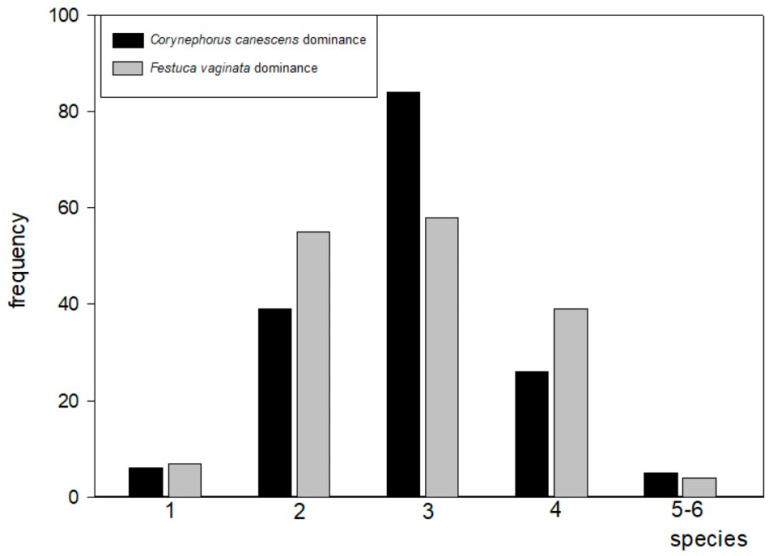
Distribution of samples among the species richness categories in the *Corynephorus canescens* and *Festuca vaginata* dominated sites, respectively, of *Festuco vaginatae–Corynephoretum* in 0.01 m^2^ size microplots.

The distribution of the samples among the species richness classes and biomass classes proved to be significantly different in the *Corynephorus canescens* and the *Festuca vaginata* dominated sites (chi^2^ tests, from *p* = 0.01 to *p* < 0.05), irrespective of the number of categories used (Figure 3, Table 3).

Two peak equations yielded statistically satisfactory results describing the relationship between the biomass and the number of species for both sites. These were the three parameter version of the Gaussian equation as *f* = *a* × *exp*(−0.5* × *((*x* − *x*_0_)/*b*)^2^) and the three parameter version of the Lorentzian equation as *f* = *a*/(1 + ((*x* − *x*_0_)/*b*)^2^), where *x* is the cryptogamic biomass (g) at 0.01 m^2^ and *f* is the species number of cryptogamic species at 0.01 m^2^, respectively, whereas *a*, *b* and *x*_0_ are parameters calculated via iterative parameter estimate functions (Table 4).

The Gaussian equation has yielded the most satisfactory estimates. The reliability of the parameter estimates proved similarly high according to the two equations and also for the regressions (between 0.05 and 0.005 for all cases), but as a trend, the reliability of the regressions for both sites proved slightly higher for the Gaussian equation than for the Lorentzian. The regression reliabilities proved to be somewhat better for the *Festuca vaginata* dominated site (*p* < 0.005) than for the *Corynephorus canescens* dominated one (*p* < 0.015). The correlation coefficients, however, proved rather weak for both equations and for both sites.

**Figure 4:**
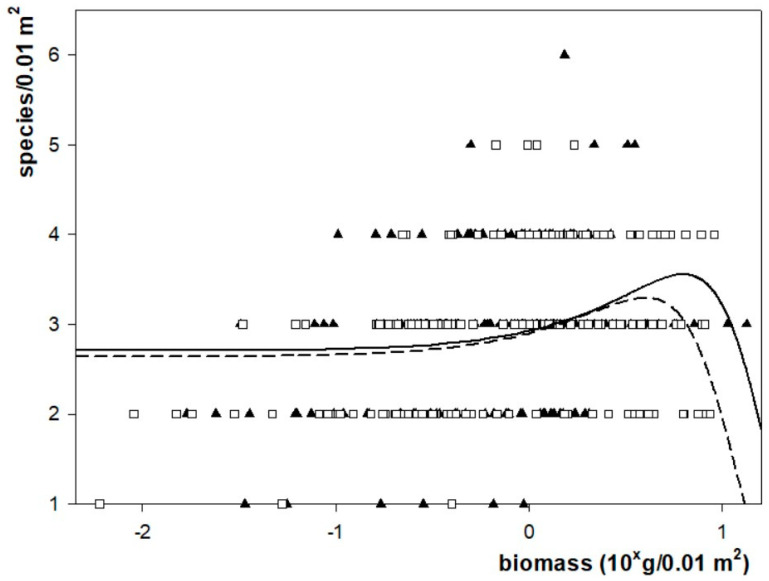
Distribution of species richness in the community as a function of the biomass of the samples taken from the *Corynephorus canescens* (filled up-triangle) and in the *Festuca vaginata* (empty square) dominated sites, respectively. The biomass scores are projected on a logarithmic scale while the species richness scores are shown on a linear scale. The used equation was a three parameter version of the Gaussian equation as *f* = *a × exp*(−0.5* × *((*x* − *x*_0_)/*b*)^2^), where *x* is the cryptogamic biomass (g) at 0.01 m^2^ and *f* is the species number of cryptogamic species at 0.01 m^2^, respectively, whereas *a*, *b* and *x*_0_ are parameters calculated via iterative parameter estimate functions. Solid line—curve calculated for *Corynephorus canescens* dominated site, dashed line—curve calculated for *Festuca vaginata* dominated site.

The values of the maxima (species richness in sample, *a* parameter) and its location along the x axis (biomass in sample, *x*_0_ parameter) have been very similarly estimated by the two equations. The estimates for the two sites differed consequently: both the maximal values and their locations proved higher in CC than in FV. The Gaussian equation, however, consequently estimated the maxima to be about 0.5% higher, irrespective of the site, while it estimated its position to be 4 to 5% higher than did the Lorentzian one (Figure 4).

### 2.3. Productivity

We made calculations of productivity (annual rate of biomass change) in the fenced parts for the two periods. Firstly, between the fence construction and the first sampling (March 2013, 4.5 years). (In this period, we considered that the biomass difference between the grazed and fenced parts has entirely been due to the effect of fencing.) Then, secondly, from the first sampling till the second one (October 2018, 5.5 years). (In this period, we used scores from the first sampling as reference.) We found that productivity altered during the study in such a way that its absolute value proved higher both for the lichen and bryophyte fractions in the second period. In the lichens, it has been increased from a rate of ≤10 g/m^2^/year for the first period to ≥20 g/m^2^/year in the second one. In the bryophytes, however, we found no consequent direction of change, as a moderately increased rate has been detected in CC in contrast to a strong decline in FV (Table 5). 

### 2.4. Diversity

The Shannon’s diversity values calculated for the frequency records (from 1.36 to 1.83) proved higher than those for the biomass ones (from 0.69 to 1.23). These figures represent evenness values from 0.54 to 0.77 (portion of possible maxima) for the frequency records and from 0.31 to 0.53 for the biomass, respectively (Table 6).

The average values of the Shannon’s diversity did not differ much between the two sites, although the averages of the evenness tended to be 6.4% (biomass) and 9.3% (frequency) higher at the FV site than at CC, respectively. For the fenced parts, the diversity scores have declined between 2013 and 2018 at both sites, whereas no congruent tendency for the unfenced parts has been detected. Decline as well as stagnating figures have been found in the grazed parts while the figures for the formerly grazed part of CC, where grazing ceased, have even increased by 2018 (Table 6). The rates of change have been more pronounced when calculated from the biomass entries.

## 3. Discussion

### 3.1. Composition and Species Richness

All the recorded cryptogams are common to the Nyírség region [77] and also to Hungary [11,78]. The only species of floristical and conservational interest is the legally protected lichen *Cladonia magyarica* Vain. [79]. Those terricolous lichen species of floristic importance which are present in the region (*Cladonia mitis* Sandst., *C. cariosa* (Ach.) Spreng., *Xanthoparmelia pokornyi* (Körb.) O. Blanco, A. Crespo, Elix, D. Hawksw. & Lumbsch and *X. pulvinaris* (Gen.) Ahti et D. Hawksw.) have not been recorded in the vicinity of our sampling sites [80,81,82]. 

As in the psammophilous grassland communities of Central Europe in general [20,25,28,63,64,65,83,84], the terricolous lichen biomass of the studied sites is dominated by various *Cladonia* species. This genus also represents a major identification challenge, and we have 10 taxa detected in the samples. It includes the morphologically similar but chemically distinct species pair *C. subulata* (only with fumarprotocetraric acid) and *C. rei* (with homosekikaic acid, ± sekikaic acid and usually with fumarprotocetraric acid) [85,86,87,88,89,90,91].

In contrast to the calcareous sands of Central Hungary with Festucetum vaginatae danubiale [92] where *C. magyarica* and *C. furcata* have been found as codominants [63,65], in our *Festuco vaginatae–Corynephoretum* samples, *C. rangiformis* has been the dominant lichen species most often. Among the bryophytes, *Syntrichia ruralis* has been the most widespread species in the calcareous sands [63], while in our samples, the bryophyte dominance has varied according to the dominant vascular plant species, management and sampling date. The dominance of *S. ruralis* has been confined to the grazed parts of both the CC and FV sites. In the fenced part of CC, *Brachythecium albicans* became dominant by 2018 while *Polytrichum piliferum* kept dominance among the bryophytes in the fenced part of FV. 

### 3.2. Biomass

The only published records of specific terricolous cryptogamic biomass from the Carpathian Basin came from IBP studies conducted on the open calcareous sandy grasslands of Central Hungary [65]. Their sampling was carried out between 1968 and 1972, although its pacing and resolution changed from time to time. The most intense periods involved monthly sampling of lichens and bryophytes from April to October in 1968 and another monthly sampling campaign of lichens between November 1970 and April 1972. Although the sampled surface on a given sampling date agreed with ours (4000 cm^2^), it only involved 10 subsamples in contrast to the 40 in our study.

The value of the cryptogamic biomass, including both the lichen and bryophyte biomass, in this study series was definitely higher than that found in our own research. While in our case the minima of the cryptogamic biomass ranged from 25–40 g/m^2^ and the maxima from 225–285 g/m^2^, in Verseghy’s studies these figures were 125–205 g/m^2^ and 260–405 g/m^2^, respectively, during the 1968 sampling campaign.

During the whole sampling period (through 29 sampling dates), only the biomass of lichens in Verseghy’s studies had minima of 50–55 g/m^2^ in both studied communities, the averages fell between 120 and 140 g/m^2^ in the Festucetum vaginatae danubiale community and between 115 and 165 g/m^2^ in the Brometum tectorum community, while the maxima reached 360 g/m^2^ and nearly 450 g/m^2^, respectively, in the order described above.

In our *Festuco vaginatae–Corynephoretum,* however, the corresponding figures for the lichens ranged from as low as 5–15 g/m^2^ for the minima while the maxima only had 155 g/m^2^ in the *Corynephorus* dominated site and 225 g/m^2^ in the *Festuca vaginata* dominated one, respectively. It is not known whether these differences are attributed to differences in the physical properties between the two landscapes or other factors.

Few data enable a direct comparison of vascular vs. cryptogamic biomass Pannonian sandy grasslands, and these are mostly from Central Hungary [63]. In open grasslands, such as Brometum tectorum, Festucetum wagneri and Festucetum vaginatae danubiale, cryptogamic biomass has been shown to exceed that of vascular plants through the vegetation period. For example, in Festucetum vaginatae, cryptogams had 170 g/m^2^ biomass in June while vascular plants had their summer peak at 150 g/m^2^. The cryptogamic surplus was even more pronounced in the other two communities, reaching up to 290 g/m^2^ biomass paralleled with only 60 g/m^2^ of vascular biomass during the June sampling [63].

In the unfenced plots of our sites, an average of (65–)80–100(–125) g/m^2^ aboveground vascular phytomass has been harvested at the beginning of the experiment. It was in contrast with (95–)110–125(–145) g/m^2^ for the fenced plots, showing an early positive effect of grazing exclosure on vascular phytomass.

In the Nyírség region, the single grassland study where biomass both of vascular plants as well as of lichens and bryophytes (none of them identified at the species level) has been sampled has recently been conducted [93]. In that work, the average vascular phytomass of sandy grasslands fell around (125–)185(–230) g/m^2^, somewhat higher than in our study. (Due to the relatively cool and humid spring weather preceding their sampling in 2021, these phytomass values are likely to fall between the average and maximal (www.met.hu accessed on 3 August 2023).) These records have been paralleled with a relatively low (0–)2.5–11(–13.5–22.5) g/m^2^ lichen biomass and (0–12.5–)23.5–62.5(–75–115) g/m^2^ bryophyte biomass, respectively.

Soil samples from these grasslands, however, had an apparently higher pH (pH_KCl_: 5.37 ± 0.46 SE) as well as higher organic matter content (*w*/*w*%: 1.44 ± 0.54 SE) compared to ours, suggesting that most samples came from more productive grasslands (e.g., Potentillo-Festucetum pseudovinae) and only some from *Festuco vaginatae–Corynephoretum*.

Minor variations in the soil properties have similar effects on the biomass at our study sites. The aboveground vascular biomass in our sites varied around (20–)55–65(–75) g/m^2^ on soils with lower organic matter content (≤0.6%), whereas it measured (110–)150–160(–185) g/m^2^ on soils relatively rich (≥1.5%) in organic matter (Matus et al. unpublished). It is very likely that above a certain amount of vascular biomass, the cryptogamic and vascular biomass are negatively correlated.

As no individual records, only group averages, are presented in [93], direct comparison neither to their vascular biomass records nor to our biomass records is possible. Within different grazing intensity classes, lichens make up averagely from 1.5 to 4.8% of the vascular biomass, whereas for bryophytes, this figure varies from 10.4 to 41.0%. The highest biomass both of lichens and bryophytes has been recorded at sites with low numbers of droppings and located far from watering places. The highest lichen biomass has been associated with the lowest stocking densities (<1 LU/ha) (LU = livestock unit; https://landmark2020.eu/glossary/livestock-unit-lsu/ accessed on 3 August 2023). This is non-experimental evidence supporting our findings concerning increased lichen biomass after grazing exclosure.

In the Nyírség region, the possible maximum of the lichen biomass could in any case be somewhat higher. On fallow lands, developed after the abandonment of acidic sandy arables, the lichen biomass can also reach up to 285 g/m^2^ (Aszalósné Balogh & Matus unpublished data). These sites are practically devoid of perennial vascular plants and have developed a low diversity, cryptogam dominated grassland where over 98% of cryptogamic biomass came from *Cladonia rangiformis* (H′: 0.1, evenness: 0.072). The rapid recovery of dense cryptogamic mats on calcareous sandy fallows in Hungary has also been described, where they can block the establishment of weed species and prevent the formation of their soil seed banks [72].

Selecting peak equations to describe the connection between the biomass and species richness biomass had the background logic of expecting a low species number both in the case of minimal biomass (e.g., in heavily grazed conditions) and in the case of high biomass (typically in a monospecific dominance situation). In contrast, for intermediate biomass scores, neither low nor high biomass seems to limit the number of species. For most of the tested equations, however, the probability of estimates for some or all parameters proved unacceptable (of high *p* values). Low correlation coefficients, even in the best-fit Gaussian and Lorentzian equations, widely accepted ones used in describing the average effect caused by a large number of factors [94], suggest that in addition to the cryptogamic biomass, a series of other factors (e.g., vascular biomass, local disturbance history, age of exclusion) might also be responsible for the small-scale cryptogamic species richness.

### 3.3. Diversity

Separation of thalli and counting ‘individuals’ can be problematic in cryptogams, so biomass can provide a more reliable basis for diversity calculations. Nevertheless, due to its time-consuming manner, specific biomass measurements have rarely been accomplished. An outstanding early example came from the analysis of dry heathland dynamics in Northern England [95], where pioneer and early degrading phases had higher diversities and evenness scores than did the intermediate ones. The figures of H′ and evenness recorded here are similar to ours (Table 6).

Although the composition of the cryptogamic assemblages of the *Corynephorus* dominated grasslands has been thoroughly studied in Central and Western Europe [22,25,26,34,84,96,97,98,99,100,101,102], specific dominances have usually been estimated using rough scales (e.g., coenological cover-abundance categories), which do not allow for a reliable calculation of diversity. The works of Verseghy [63,64,65], with an identical sampling surface to our experiment, however, enabled comparison of the diversities of lichen biomass records (no specific bryophyte biomass scores have been published by her).

We found that the lichen diversity and especially the evenness of the biomass samples from Festucetum vaginatae danubiale (H′: 1–1.5, evenness: >0.6) greatly exceed those of our samples from *Festuco vaginatae–Corynephoretum* (H′: 0.04–0.94, evenness: <0.41). Even the diversities calculated for Verseghy’s Brometum tectorum samples proved somewhat higher (H′: 0.44–0.9, evenness: 0.4–0.9). In our study, the lowest H′ and evenness values have been associated with long-term grazing exclosure (in 2018), both in CC and FV (Table 7).

We found that the lichen diversity of the fenced parts already started to decline before the first sampling (4.5 years after exclosure) and dropped greatly till the second sampling (10 years after exclosure). These changes can be well interpreted by the ‘intermediate disturbance theory’ [103,104]. In our case, ‘intermediate disturbance’ can mean rare, low-intensity grazing which prevents *Cladonia rangiformis* from becoming monodominant. Less competitive, smaller and/or short-lived cryptogams can, however, colonize neither under heavy grazing pressure nor in the total absence of grazing. 

### 3.4. Management

Lichens and bryophytes possess a more fragile structure and have slower growth rates than most of the vascular plants [105]. They can be used in the evaluation of grassland management practices or can even serve as differential species in grassland classification [52]. One can find different views in the literature on the impact of grazing on cryptogams. Studies where no discernible effect was found are, however, quite rare (alpine communities in the Scottish Highlands; [61]). Mostly, profound effects of grazing both on biomass and composition have been reported by Scandinavian authors [50,51,52,53,106,107,108]. Understanding the impacts of grazing is further complicated by the fact that in most cases, the direct and indirect effects of grazing on cryptogams have not been separated, mainly because the experimental design did not allow it.

The first direct effect of grazing on cryptogams is the consumption, although its importance also depends on the grazer. Consumption of lichen by reindeer has strong seasonal constraints; nevertheless, it forms an important part of the reindeers’ winter diet [61,109]. No apparent literature source on lichen consumption by cattle is available, and there is limited information on the cattle’s bryophyte consumption too, although a significant reduction of moss cushions is reported in alpine grasslands [110]. Sheep are known for consuming lichens, though a study in Norwegian subalpine grasslands proved that only less than 1% of their diet comes from this source [111,112]. According to interviews with shepherds in our study region, the consumption of lichen by sheep is negligible.

In contrast to the often low impact of consumption, the decline of lichen biomass and species richness can rather be linked to trampling in various ecosystems. The effect of trampling seems important in the arctic landscape [56,113] but tends to be more profound in drylands, for example, in semi-deserts (e.g., the western United States [52]), as cryptogams spend most of the year in an inactive, brittle state. By similar logic, trampling, instead of consumption, seems to be the decisive direct effect in our continental study sites.

In dry conditions, cryptogams are more sensitive to trampling [114]. Humidity was found to be an important factor used to predict lichens’ trampling loss [115]. Trampling can also have a species-specific effect on bryophytes. While most species are sensitive to it, *Polytrichum hyperboreum* in arctic grasslands was found to be resistant to trampling even during dry summers [106]. Different grazing densities can have contrasting effects on the reproduction of lichens, as while heavy grazing can lead to the extinction of populations, moderate grazing can actually help the species to reproduce from fragments [75]. This can be the case for *C. magyarica* Vain., the only lichen species favored by moderate (fragmenting) grazing then abandonment. Short spells of low-intensity grazing seem to promote this legally protected species in the studied grasslands. Some bryophytes also showed a rapid recovery after grazing exclusion, probably due to their ability to re-establish from fragments and to tolerate the shading caused by plant cover [44]. Generalization can be misleading due to the high variability of species-specific responses [116,117]. Further studies are necessary to elucidate how the effects of grazing and trampling vary by lichen growth forms and moss strategy types [118].

Grazing, however, can have an indirect impact on cryptogams, too. On the one hand, removing vascular biomass can promote the development of cryptogams via decreased shading. It was concluded that cryptogams in heavily grazed parts are typical fugitive species which cannot stand competition in more dense vegetation [52]. Additionally, *Polytrichum* species and small *Cladonia* species with non-branching podetia also possess apparently effective dispersal mechanisms, which enables them to colonize areas devoid of competitive large, branching *Cladonia* species [62]. The issue of interspecies competition can sometimes be more complex, as [119] have found a significant decline in the cover of lichens and some bryophytes as the grazing impact increased along with a positive effect of vascular plant cover on cover of other bryophytes. This response was similar to that seen in many other studies [44,47,48,49].

**Figure 5:**
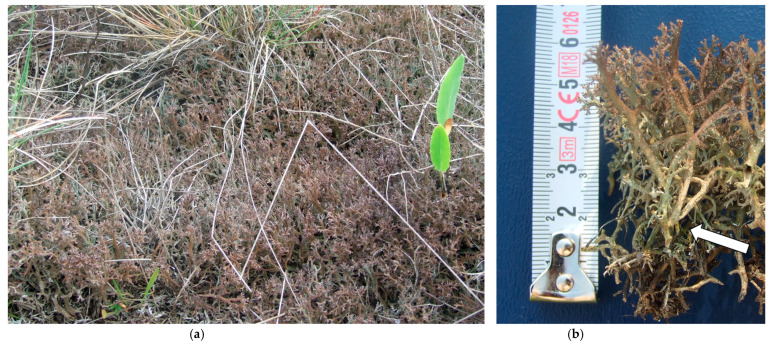
(**a**) Masses of *Cladonia rangiformis* limiting growth of vascular plants (note the stunted specimens of *Eryngium campestre* and *Rumex acetosella)*, and (**b**) cross-section of a >60 mm thick mat of *C. rangiformis* (note the single stem of *Brachythecium albicans* indicated by the arrow). Both photos taken at a fenced part of the *Festuca vaginata* dominated site, May 2018.

Competition among cryptogams and between cryptogams and vascular plants can not only be described by the biomass distribution but also by the height of the species involved [120]. In the absence of grazing, successful cryptogamic competitors tend to be tall species, either bryophytes (such as *Herbertus hutchinsiae* Gottsche & Rabenh. in a study from Scotland) [117] or more often lichens, especially various large species of *Cladonia* [56,116]. In line with this, 50 to 60 mm tall mats of *C. rangiformis*, one of the tallest lichens in our study, can already effectively block smaller cryptogams and even hinder the growth of most vascular plants (Figure 5). In contrast, bryophytes lost their dominance between 2013 and 2018, either due to inferior growth compared to lichens (and maybe to exclosure-promoted vascular plants) (CC) or even in absolute figures (FV).

Increasing grazing intensity has been shown to consistently lead to a decline in cryptogams’ richness, while the number of lichens and bryophytes increased with the time since exclusion in drylands [44,45,52]. Grazing triggers opposite processes, and the results of our experiment are consistent with this. It is not known, however, how this effect is shared among sheep and cattle (both the cattle and sheep grazing intensity changed during the study period) or to what extent does this effect come from the lack of herbivory or of trampling. The literature, however, supports a bigger effect of the latter [75,110,121].

Although there is a consensus that biomass accumulation during cryptogamic (primary) succession can be very slow, lasting for decades [6,65,122,123,124], a few years of fencing in our study has already led to an increased biomass in the secondary dynamics of the cryptogams. In differently aged exclosures, the responses of cryptogams can also change with time and vary specifically. The lichen cover as well as total cryptogamic cover increased (the latter from 4% to 15%) in the midterm grazing exclosures (14–18 years) but declined in the long-term (37–38 years), while the moss cover continued to increase with time [52]. We also detected productivity changes in different phases of the exclosure-induced dynamics (Table 5) and revealed changing growth rates through a primary cryptogamic succession [124], although we were still not able to predict a species-specific outcome in continuation of our experiment. 

A further challenge is to investigate changes in vascular plants, bryophytes and lichens simultaneously in our grazing exclosures. Such a complex approach, covering the spatial and diversity constraints of all of these groups, is very rare in the literature [6,125,126]. Similarly, learning more about the effect of the cryptogamic layer on the establishment of vascular plants [72] is important to gain a more detailed insight into the community dynamics of managed grasslands. A further factor worth studying is how the nutrient input from the dung of ungulate grazers can promote vascular plant growth and therefore affect cryptogamic biomass indirectly. Similarly, this factor can change the competition among cryptogamic species likewise.

## 4. Materials and Methods

### 4.1. Characteristics of the Study Area

We studied the dynamics of cryptogams (bryophytes, lichens) in two dry sandy grassland sites in East Hungary, located 15 km and 25 km east of the city of Debrecen, respectively. Both belong to the G1 class of national vegetation classification, A-NÉR *Open sand steppes*. A site of *Corynephorus canescens* dominance (CC, 47°31′55.97″ N, 21°56′59.40″ E, 128 m a.s.l.) and another one of *Festuca vaginata* dominance (FV, 47°34′30.05″ N 21°47′31.84″ E, 129 m a.s.l.), phytosociologically belonging to the grassland community *Festuco vaginatae–Corynephoretum* Soó in Aszód 1935 [127], have been monitored. This community inhabits dunes of the calcium poor sands of the Nyírség geographical region *(*https://novenyzetiterkep.hu/node/765 accessed on 29 May 2023) and is classified by Natura 2000 habitats as ‘6260 Pannonic sand steppes’ (closely related to 2340 ‘Pannonic inland dunes’) (https://natura2000.eea.europa.eu/ accessed on 29 May 2023). 

In addition to their dominant grasses, providing the phytosociological name of the community, these sites host a number of further frequent monocots, such as *Carex stenophylla*, *Cynodon dactylon* and *Poa bulbosa*. Their perennial dicots with significant cover include *Chondrilla juncea*, *Eryngium campestre*, *Potentilla arenaria*, *Rumex acetosella*, and *Thymus degenianus*. (The underlined species as well as *Festuca vaginata* at first benefited being excluded from grazing.) Further constituents of these communities are the horsetail *Equisetum ramosissimum* as well up to about 30 further angiosperms, mostly small annual dicots. These communities are subject to different levels of grazing from domestic herbivores (mostly beef cattle and/or sheep) and have become increasingly abandoned in the latest decades due to dropping livestock densities.

Cryptogamic vegetation has been compared at both sites in 2013 and 2018, respectively, in an experimentally fenced part (a 5 m by 25 m size plot within the exclusion where the grazing of domestic animals has been excluded since summer of 2008) as well as a non-fenced part subject to changing grazing pressures, respectively. The fenced and unfenced parts are located at 20 to 30 m apart and had a similar composition and dominance when fencing has taken place. Fencing was used to model the prevailing trend of falling stocking densities.

At the CC site, a flock of about 140 sheep grazed during the growing season, passing through twice the area per day. Grazing, however, has been ceased in late 2015 as the forestry directorate, the owner of the area, has no longer contributed to it in order to protect new forest plantations nearby.

The FV site was mainly grazed by a herd of around 40 beef cattle, less frequently by around 70 to 100 sheep. Grazing here has fluctuated, but its intensity has shown an overall increase throughout the study period. Hares and roe deer have been observed accidentally in the non-fenced parts of both communities, although the low density of their droppings suggested only a negligible grazing pressure. Therefore, the 140 cm tall fence, excluding sheep, cattle roe deer and hare, almost completely eliminated the effect of grazing. It was not possible, however, to exclude small rodents. We did not separate the effects of cattle and sheep, nor was it possible to separate the effects of grazing (biomass removal) and trampling (causing biomass decay).

The climate of the study areas is moderately continental, with hot and dry summers (precipitation is mainly caused by thunderstorms and downpours), while winters are a few degrees colder and harsher than the national average. The average of the annual temperature is around 10.5 °C, while the annual sum of sunshine exceeds 2200 h. The average of the yearly precipitation falls between 550 and 600 mm. The average of the rainfall during the vegetation period (April−September) is between 300 and 350 mm, with extremes from 210 to 620 mm ([128], https://www.met.hu/eghajlat/ accessed on 29 May 2023).

At the beginning of the experiment, the vascular phytomass in the *Festuca vaginata* dominated site fell around (65–)80–120(–125) g/m^2^ while that of the *Corynephorus canescens* dominated one was (95–)100–120(–145) g/m^2^, respectively (Matus et al. unpublished). Production of vascular phytomass varied yearly following weather patterns, with the highest figures in 2008 and the lowest ones in 2009, respectively. The average production in the unfenced stands has stayed at 80–100 g/m^2^ while that of fenced ones has been at around 120 g/m^2^ in first three years of exclosure, respectively. 

The topsoil (0–5 cm) of the sites is unanimously sand (portion of the 0.05–2 mm size fraction is 93.06% in CC and 95.36 in FV) of acidic chemistry (pH_KCl_–CC: 4.44–4.45; pH_KCl_–FV: 4.80–4.84) and with negligible CaCO_3_ content (at both sites: <0.05 *w*/*w*%), low organic matter content (CC: 1.09–1.34%; FV: 0.82–1.00%) and low available nitrogen (NO_2_ + NO_3_–N–CC: 2.58–4.55 ppm; NO_2_ + NO_3_–N–FV: 1.08–2.67 ppm) as well as low phosphorous (AL-P_2_O_5_–CC: 34–55 ppm; AL-P_2_O_5_–FV: 26–43 ppm) content. Somewhat higher figures for organic matter and phosphorous in CC can be indicators of the former arable usage decades before the beginning of the experiment.

### 4.2. Biomass Sampling

The biomass of the cryptogams has been sampled in 2013 and in 2018 (four and a half and ten years after the exclosure, respectively). The aboveground vegetation of 40, 10 × 10 cm size (0.01 m^2^), microplots per site per management have been collected with a soil monolith between 25 February and 7 March in 2013 and between 10 and 14 October 2018, respectively. The microplots have been (1) evenly scattered in zones along the length and width of plots, so as their middle and ends as well as their center and sides closer to the bordering buffer zone are similarly represented and (2) have been centered on randomly thrown markers (within selected zones) in order to guarantee a similar representation of grass tussocks and gaps.

The microplots with underlying soil monoliths of 5 cm depth have been transferred to the lab, where the samples were dried then hand-sorted into bryophyte and lichen species under a stereo microscope, and these dry fractions were measured with an accuracy of 0.001 g.

A preliminary survey of the vascular biomass of the study sites has been completed at the beginning of the exclusion experiment. Twenty samples, each of 20 × 20 cm size (0.04 m^2^), have been collected yearly from blocks at the end of June between 2008 and 2010. This period involved a rather dry year (2009, with very dry April and May and 0.5 °C higher than average temperature in March–June) as well as a very wet year (2010, with an exceptionally rainy March-June period and a temperature somewhat under the mean) and so represents a large portion of the possible weather fluctuations in the region (www.met.hu accessed on 29 May 2023).

### 4.3. Soil Analysis

Soil analyses have been carried out by the accredited laboratory of the Research Institute of Karcag of the Hungarian University of Agriculture and Life Sciences. The following three parameters were measured: pH (KCl), total organic matter content (*m*/*m*%) and available phosphorus (mg/kg). In the case of the pH, we have used the potentiometric method and have measured the parameter in a potassium chloride solution suspension. The organic matter content has been determined via the Tyurin method, so we have used the titrimetric method and oxidation by chromic acid. The phosphorus content of the soil extract has been converted into heteropoly acid with ammonium molybdate and has been reduced to a molybdenum blue complex with ascorbic acid. The AL-soluble phosphorus (AL–P_2_O_5_) content of the soils was determined via spectrophotometry. The light absorption has been measured at 480 nm.

### 4.4. Identification

Lichen identification has been completed using the manuals of Verseghy [78], Smith et al. [129] and Wirth et al. [91]. The nomenclature of lichens mostly follows the public database of Index Fungorum (http://www.indexfungorum.org/, accessed on 29 May 2023) and the public database of MycoBank (https://www.mycobank.org/, accessed on 29 May 2023). For the identification of critical taxa in the lichen genus *Cladonia*, standard spot tests and high-performance thin-layer chromatography (HPTLC) in solvent C (toluene: acetic acid = 20:3, V/V) have been applied [130]. The latter has been used in order to determine the pattern of secondary metabolites, an inevitable trait for identification in the genus. Approximately 5 mm × 5 mm air-dried thallus fragments were soaked in 0.2 mL acetone for 30 min in order to extract the lichen substances. Pretreated (50 °C for 5 min, CAMAG TLC Plate Heater III, then cooled to room temperature) 10 cm × 10 cm thin-layer chromatographic plates (Merck, Kieselgel 60 F254) were used. A total of 8 µL acetone extracts (1 µL at each time) were applied to each position (5 mm apart) on the plate using a microapplicator (CAMAG Micro Applicator I). *Pleurosticta acetabulum* (Neck.) Elix & Lumbsch (norstictic acid) and *Leucodermia boryi* (Fée) Kalb (atranorin, zeorin) were used as controls. After chromatographic development, the plates were examined under UV light (254 and 366 nm) then sprayed (CAMAG TLC Sprayer) with water, dried (to investigate the fatty acids), then subsequently sprayed with a 10% sulfuric acid solution and heated at 110 °C for 5–10 min. Finally, the plates were cooled to room temperature and studied under UV light (366 nm).

Bryophytes have been identified by consulting the relevant handbooks and papers according to Erzberger and Schröder [131], while the nomenclature for bryophytes follows Hodgetts et al. [132]. A Wild dissecting microscope and a Leica light microscope were used.

### 4.5. Data Processing

Pairwise and multiple comparisons have been performed using the SigmaPlot 12.0 software package. Comparisons of the species number and biomass data per site per management per date have been performed via ANOVAs on the ranks, and in case of a significant result, we used Kruskal–Wallis tests to identify a significant pairwise difference(s).

Principal coordinate analysis (PCA) supported by the CANOCO and CANODRAW software packages [133] has been applied to analyze and to visualize the quantitative relations of the samples both for the frequency and biomass records, respectively. Input data have been gained by pooling 40 subsamples per site per treatment per date so 8 points of pooled data have been projected. Species present in less than three pooled samples have been excluded from the analyses.

The possible correlation of the cryptogamic biomass and the combined species number of lichens and bryophytes has been also studied. After a preliminary evaluation of the data, we systematically tested the available peak equations supported by the SigmaPlot 12.0 software package (Gaussian with 3 and 4 parameters, modified Gaussian with 4 and 5 parameters, Lorentzian with 3 and 4 parameters, Pseudo-Voigt with 4 and 5 parameters, log normal with 3 and 4 parameters and Weibull with 4 and 5 parameters, respectively). We used the package option ’dynamic fitting’ enabling estimation of parameters via an iterative approach so as to obtain the best possible estimates for the parameters and to achieve the best possible fit. The automatic initial parameter estimate function has been used as well as a 200 fit option, while the maximal number of iterations has also been set to 200. The accuracy (probability) of the parameter estimates, results of the analysis of variance (ANOVA, corrected for the mean of the observations) for the rates of regression and residual as well as the magnitude of the correlation coefficients have been evaluated.

We compared the *Corynephorus canescens* and *Festuca vaginata* dominated sites by the distribution of the samples among the species numbers classes as well as among the biomass classes using chi^2^ tests [94]. These tests have been applied on two setups using three (df = 2) and five categories (df = 4), respectively. For species richness in three categories as ≤2, 3, ≥4 species and in five categories as 1, 2, 3, 4, ≥5 species/0.01 m^2^, while for biomass records in three categories as <0.5, 0.5–1, ≥1 g and in five categories as <0.1, 0.1–0.5, 0.5–1, 1–5, ≥5 g dry biomass/0.01 m^2^. 

Shannon’s diversity and evenness [94,134] have been calculated for specific frequencies and specific biomass scores after pooling data gained from 40 microplots, respectively, on the 2 sampling dates in differently managed parts of the *Corynephorus canescens* (CC) and *Festuca vaginata* (FV) dominated sites. The maximal possible diversity values have been calculated using the total number of taxa recorded throughout the study at a given site and an even dominance distribution.

## Figures and Tables

**Table 1:** Average numbers of lichen and bryophyte taxa and all cryptogams, respectively, on different sampling dates in differently managed parts of the studied communities (*n* = 40). (CC—*Corynephorus canescens* dominance, FV—*Festuca vaginata* dominance). Values exhibiting different letters indicate significant differences in the medians according to Tukey tests. Read vertically and interpret within a community and a taxonomic group (e.g., among lichens in CC).

Site, Year, Management	Mean ± SE		
	Lichens	Bryophytes	Cryptogams
CC 2013 grazed	0.85 ± 0.11 b	1.98 ± 0.09 a	2.83 ± 0.15 b
CC 2013 fenced	1.75 ± 0.20 a	1.75 ± 0.08 ab	3.50 ± 0.24 a
CC 2018 grazing ceased	1.35 ± 0.13 ab	1.60 ± 0.09 b	2.95 ± 0.14 ab
CC 2018 fenced	1.20 ± 0.09 b	0.48 ± 0.08 ab	2.98 ± 0.10 ab
H-value	18.47	10.88	5.63
*p*-value	<0.001	0.01	0.13
FV 2013 grazed	1.03 ± 0.08 b	2.45 ± 0.14 a	3.48 ± 0.19 a
FV 2013 fenced	1.45 ± 0.09 a	0.73 ± 0.12 b	3.43 ± 0.16 a
FV 2018 grazed	1.13 ± 0.06 b	1.23 ± 0.08 c	2.35 ± 0.12 b
FV 2018 fenced	1.53 ± 0.09 a	1.60 ± 0.09 bc	3.13 ± 0.14 a
H-value	25.42	49.00	32.18
*p*-value	<0.001	<0.001	<0.001

**Table 2:** Average biomass of lichen and bryophyte taxa and average of all cryptogams, respectively, on different sampling dates in differently managed parts of the studied communities (*n* = 40). (CC—*Corynephorus canescens* dominance, FV—*Festuca vaginata* dominance). Values exhibiting different letters indicate significant differences in the medians according to Tukey tests. Read vertically and interpret within a community and a taxonomic group (e.g., among lichens in CC).

Site, Year, Management	Mean ± SE (g/0.01 m^2^)		
	Lichens	Bryophytes	Cryptogams
CC 2013 grazed	0.06 ± 0.01 b	0.35 ± 0.07 a	0.41 ± 0.07 b
CC 2013 fenced	0.42 ± 0.10 b	0.47 ± 0.08 a	0.89 ± 0.11 b
CC 2018 grazing ceased	0.42 ± 0.12 b	0.72 ± 0.09 a	1.14 ± 0.12 b
CC 2018 fenced	1.58 ± 0.47 a	0.68 ± 0.14 a	2.26 ± 0.46 a
H-value	28.70	12.26	36.87
*p*-value	<0.001	0.01	<0.001
FV 2013 grazed	0.16 ± 0.03 b	1.05 ± 0.15 b	1.21 ± 0.16 b
FV 2013 fenced	0.59 ± 0.09 b	2.25 ± 0.33 a	2.84 ± 0.39 a
FV 2018 grazed	0.07 ± 0.01 b	0.17 ± 0.03 c	0.24 ± 0.03 b
FV 2018 fenced	2.08 ± 0.37 a	0.63 ± 0.14 bc	2.70 ± 0.39 a
H-value	67.25	55.37	63.15
*p*-value	<0.001	<0.001	<0.001

**Table 3:** Comparison of *Corynephorus canescens* and *Festuca vaginata* dominated sites by the distribution of samples between the species richness classes and biomass classes, respectively. For species richness in three categories as ≤2, 3, ≥4 species and in five categories as 1, 2, 3, 4, ≥5 species/0.01 m^2^, while for biomass records in three categories as <0.5, 0.5−1, ≥1 g and in five categories as <0.1, 0.1−0.5, 0.5−1, 1−5, ≥5 g dry biomass/0.01 m^2^.

		chi^2^	*p*-Value
5 categories (df = 4)	Species richness	10.595	0.032
	biomass	11.917	0.018
3 categories (df = 2)	Species richness	8.591	0.014
	biomass	9.188	0.010

**Table 4:** Correlation of cryptogamic biomass and species richness at the 0.01 m^2^ scale at *Corynephorus canescens* (CC) and *Festuca vaginata* (FV) dominated sites, respectively, according to two peak equations, the three parameter versions of the (**a**) Gaussian and (**b**) Lorentzian equations, respectively. Results of the ANOVA (corrected for the mean of observations) showing the rate of regression and that of the residual by the above equations and parameters calculated via iterative parameter estimate functions as well probability of these estimates. ni—number of iterations, df—degrees of freedom, SS—Sum of Squares, MS—Mean Square, F—critical value, *p*—probability, SE—Standard Error, *t*—t value, ratio of parameter and SE.

(**a**)											
**r_CC_ = 0.2336** **ni: 10**	**ANOVA**	**df**	**SS**	**MS**	**F**	***p***		**Parameter**	**SE**	**t**	***p***
	**regression**	2	6.154	3.077	4.5313	0.0122	***a***	3.555	0.263	13.526	<0.0001
	**residual**	157	106.621	0.679			***b***	8.456	1.822	4.642	<0.0001
	**total**	159	112.775	0.709			***x*_0_**	6.270	1.237	5.069	<0.0001
**r_FV_ = 0.2648** **ni: 12**	**ANOVA**	**df**	**SS**	**MS**	**F**	***p***		**Parameter**	**SE**	**t**	***p***
	**regression**	2	9.058	4.529	5.9184	0.0033	***a***	3.293	0.144	22.938	<0.0001
	**residual**	157	120.136	0.765			***b***	5.966	1.011	5.901	<0.0001
	**total**	159	129.194	0.813			***x*_0_**	3.998	0.48	8.325	<0.0001
(**b**)											
**r_CC_ = 0.2295** **ni: 15**	**ANOVA**	**df**	**SS**	**MS**	**F**	***p***		**Parameter**	**SE**	**t**	***p***
	**regression**	2	5.938	2.969	4.363	0.014	***a***	3.537	0.278	12.724	<0.0001
	**residual**	157	106.837	0.681			***b***	10.825	2.631	4.115	<0.0001
	**total**	159	112.775	0.709			***x*_0_**	5.956	1.286	4.630	<0.0001
**r_FV_ = 0.2582** **ni: 9**	**ANOVA**	**df**	**SS**	**MS**	**F**	***p***		**Parameter**	**SE**	**t**	***p***
	**regression**	2	8.614	4.307	5.608	0.004	***a***	3.277	0.147	22.244	<0.0001
	**residual**	157	120.580	0.768			***b***	7.939	1.536	5.169	<0.0001
	**total**	159	129.194	0.813			***x*_0_**	3.842	0.502	7.652	<0.0001

**Table 5:** Productivity (rate of biomass change, g/m^2^/year) of the lichen and bryophyte fractions in the fenced parts of the *Corynephorus canescens* dominated site (CC) and in the *Festuca vaginata* dominated (FV) site of *Festuco vaginatae–Corynephoretum* between the fence construction and the first sampling and between the first and second sampling, respectively.

Period	CC		FV	
	Lichens	Bryophytes	Lichens	Bryophytes
July 2008–March 2013	8.4	2.2	10.0	26.7
March 2013–October 2018	20.1	3.9	26.6	−29.5

**Table 6:** Cryptogamic Shannon’s diversity and evenness calculated for specific frequencies and specific biomass scores of the samples, respectively, based on data from the two sampling dates at differently managed parts of the *Corynephorus canescens* (CC) and *Festuca vaginata* (FV) dominated sites of *Festuco vaginatae–Corynephoretum.* Maximal possible diversity values have been calculated using the total number of bryophyte and lichen taxa recorded throughout the study in the given site (15 taxa in CC and 9 taxa in FV, respectively) and an even dominance distribution.

Shannon’s Diversity		CC				FV			
		2013	2013	2018	2018	2013	2013	2018	2018
		Grazed	Fenced	Grazing Ceased	Fenced	Grazed	Fenced	Grazed	Fenced
**Frequency**	**H′**	1.593	1.838	1.679	1.443	1.628	1.681	1.365	1.601
	**Evenness**	0.6038	0.6963	0.6363	0.5467	0.741	0.765	0.6214	0.7286
**Biomass**	**H′**	1.059	1.231	1.098	0.828	1.147	1.082	1.156	0.689
	**Evenness**	0.4012	0.4664	0.4159	0.3138	0.522	0.4923	0.5261	0.3136
	**Max.**	**2.639**				**2.197**			

**Table 7:** Comparison of the lichen diversity and evenness of the biomass samples from dry *Calluna* heathlands in Northern England [95], Brometum tectorum and Festuco vaginatae danubiale of Hungary [65] as well as fenced parts of *Festuco vaginatae–Corynephoretum* (this study) calculated from pooled samples from an identical total surface area.

	**Dry *Calluna* heathland, Yorkshire, Coppins and Shimwell (1971)**			
	**Pioneer**	**Building**	**Mature**	**Mature/degrading**
H′	1.326	0.402	0.346	0.607
Evenness	0.5757	0.1746	0.1503	0.2638
	**Brometum tectorum, Csévharaszt, Verseghy (1977)**			
	**03.1971**	**10.1971**	**03.1972**	**10.1972**
H′	0.783	0.435	0.653	0.902
Evenness	0.7125	0.3960	0.5948	0.8208
	**Festucetum vaginatae danubiale, Csévharaszt, Verseghy (1977)**			
	**03.1971**	**10.1971**	**03.1972**	**10.1972**
H′	1.015	1.505	1.177	1.087
Evenness	0.6306	0.9352	0.7310	0.6754
	**Festuco vaginatae–Corynephoretum, Nyírség, this study**			
	**03.2013**			
	**CC grazed**	**CC fenced**	**FV grazed**	**FV fenced**
H′	0.941	0.362	0.316	0.239
Evenness	0.4085	0.1572	0.1963	0.1484
	**10.2018**			
	**CC grazing ceased**	**CC fenced**	**FV grazed**	**FV fenced**
H′	0.673	0.101	0.299	0.040
Evenness	0.2922	0.0437	0.1861	0.0248

## Data Availability

Data are contained within the article.

## Appendix A

**Table A1:** The whole taxon dataset in the *Corynephorus canescens* dominated site on both dates with their frequencies and mean biomass data.

*Corynephorus canescens* Dominance								
	2013				2018			
Lichens	Frequency		Mean Biomass (g/m^2^)		Frequency		Mean Biomass (g/m^2^)	
	Grazed	Fenced	Grazed	Fenced	Grazing Ceased	Fenced	Grazing Ceased	Fenced
*Cladonia fimbriata* (L.) Fr.	0	2	0.00	0.31	0	0	0.00	0.00
*Cladonia furcata* (Huds.) Schrad.	3	9	0.48	0.84	1	2	0.05	0.04
*Cladonia magyarica* Vain.	9	2	1.65	0.02	13	3	30.32	2.68
*Cladonia pyxidata* (L.) Hoffm.	0	1	0.00	0.01	0	0	0.00	0.00
*Cladonia rangiformis* Hoffm.	19	34	3.62	40.31	28	38	9.57	154.49
*Cladonia rei* Schaer.	1	15	0.04	1.38	11	5	1.11	0.26
*Cladonia* sp.	1	3	0.02	0.10	0	0	0.00	0.00
*Cladonia subrangiformis* L. Scriba ex Sandst.	0	3	0.00	0.40	0	0	0.00	0.00
*Cladonia subulata* (L.) Weber ex F.H. Wigg.	0	0	0.00	0.00	1	0	0.02	0.00
*Diploschistes muscorum* (Scop.) R. Sant.	1	1	0.03	0.07	0	0	0.00	0.00
**Altogether**	**34**	**70**	**5.83**	**43.42**	**54**	**48**	**41.07**	**157.47**
**Bryophytes**								
*Brachythecium albicans* (Hedw.) Schimp.	37	39	9.72	20.74	22	38	1.35	55.48
*Ceratodon purpureus* (Hedw.) Brid.	1	0	0.01	0.00	0	0	0.00	0.00
*Polytrichum piliferum* Hedw.	3	1	0.06	0.11	4	2	4.14	0.07
*Syntrichia ruralis* (Hedw.) F. Weber & D. Mohr	38	30	25.47	25.92	38	31	66.68	12.42
**Altogether**	**79**	**70**	**35.25**	**46.77**	**64**	**71**	**72.17**	**67.97**
**All cryptogams**	**113**	**140**	**41.08**	**90.19**	**94**	**125**	**113.24**	**225.44**

**Table A2:** The taxon dataset in the *Festuca vaginata* dominated site on both dates with their frequencies and mean biomass data.

*Festuca vaginata* Dominance								
	2013				2018			
Lichens	Frequency		Mean Biomass (g/m^2^)		Frequency		Mean Biomass (g/m^2^)	
	Grazed	Fenced	Grazed	Fenced	Grazed	Fenced	Grazed	Fenced
*Cladonia foliacea* (Huds.) Willd. (syn.: *C. convoluta* (Lam.) Anders)	3	2	1.02	1.54	5	2	0.66	0.46
*Cladonia furcata* (Huds.) Schrad.	2	0	0.29	0.00	0	0	0.00	0.00
*Cladonia rangiformis* Hoffm.	36	39	15.43	57.79	38	39	7.42	206.71
*Cladonia rei* Schaer.	0	15	0.00	0.57	2	20	0.02	0.76
*Cladonia subrangiformis* L. Scriba ex Sandst.	0	1	0.00	0.77	0	0	0.00	0.00
**Altogether**	**41**	**57**	**16.45**	**61.57**	**45**	**61**	**8.10**	**207.93**
**Bryophytes**								
*Brachythecium albicans* (Hedw.) Schimp.	33	23	3.81	6.62	2	26	0.02	3.71
*Ceratodon purpureus* (Hedw.) Brid.	6	1	0.15	0.46	0	0	0.00	0.00
*Polytrichum piliferum* Hedw.	28	30	46.70	170.87	18	26	5.18	53.72
*Syntrichia ruralis* (Hedw.) F. Weber & D. Mohr	31	25	54.21	47.13	29	12	11.28	5.23
**Altogether**	**98**	**79**	**104.87**	**225.08**	**49**	**64**	**16.48**	**62.66**
**All cryptogams**	**139**	**136**	**121.32**	**286.65**	**94**	**125**	**24.58**	**270.59**
